# Microalgae and Macroalgae as Advanced Sources of Tyrosinase Inhibitors

**DOI:** 10.3390/molecules31010020

**Published:** 2025-12-20

**Authors:** Joanna Harasym, Katarzyna Hałdys

**Affiliations:** 1 Department of Biotechnology and Food Analysis, Wroclaw University of Economics and Business, Komandorska 118/120, 53-345 Wroclaw, Poland; 2 Adaptive Food Systems Accelerator-Science Centre, Wroclaw University of Economics and Business, Komandorska 118/120, 53-345 Wroclaw, Poland; 3 Department of Chemical Technology, Wroclaw University of Economics and Business, Komandorska 118/120, 53-345 Wroclaw, Poland; katarzyna.haldys@ue.wroc.pl

**Keywords:** tyrosinase, macroalgae, seaweed, melanin, melanogenesis, enzyme inhibition, phlorotannins

## Abstract

Tyrosinase (EC 1.14.18.1) is the primary enzyme responsible for melanogenesis in mammals and enzymatic browning in food, creating a high demand for potent, safe inhibitors of this enzyme in the cosmetic, medical, and agricultural sectors. Conventional synthetic inhibitors often face limitations concerning their cytotoxicity and stability, necessitating the exploration of marine natural products (MNPs). Marine algae, comprising macroalgae (seaweeds) and microalgae (including cyanobacteria), represent an underexploited source of structurally diverse bioactives. Macroalgae, particularly brown species, yield complex phlorotannins, such as the non-competitive oligomer dieckol, which exhibits an IC50 of 2.16 µg/mL. Conversely, microalgae deliver high-potency, low-molecular-weight compounds, notably the synthesizable scytonemin monomer (ScyM) with an IC50 of 4.90 µM—significantly stronger than kojic acid. Mechanistic analysis, supported by molecular docking, reveals diverse modes of action, from the two-step slow binding of complex phlorotannins to the highly specific competitive binding of red algal bromophenols. Translational success requires the consistent application of green extraction techniques, such as Natural Deep Eutectic Solvents (NADESs), and advanced delivery systems, like Nanostructured Lipid Carriers (NLCs), to ensure the stability and bioavailability of these compounds for future cosmeceutical and medical applications.

## 1. Introduction

### 1.1. Tyrosinase

Tyrosinase (EC 1.14.18.1) is an oxidoreductase enzyme [1]. This metalloprotein contains two copper ions in its active site, which are essential for its activity, and also activates O_2_ during the catalytic process [2], classifying tyrosinase as a type-3 metalloprotein. The reactions catalyzed by tyrosinase are initial key steps in melanin biosynthesis that make them a rate-limiting stage in melanogenesis.

**Figure 1:**
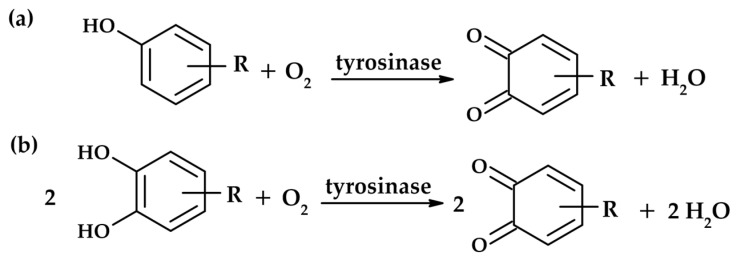
Reactions catalyzed by tyrosinase: monophenolase activity (**a**) and diphenolase activity (**b**).

Tyrosinase is able to catalyze two distinct reactions (Figure 1): the oxidation of monophenols to o-quinones (monophenolase activity) and the oxidation of o-diphenols to o-quinones (diphenolase activity) [3]. Both activities arise from the binding of the dioxygen molecule to the two copper ions (CuA and CuB) located in the active site of the enzyme [4].

Melanins are natural pigments widely distributed in nature. They differ in origin (multiple substrates) and structure. In general, melanins are the product of a complex biosynthetic pathway called melanogenesis, which occurs in organisms from bacteria to mammals. This process has been intensively studied for a long time [5,6,7,8].

**Figure 2:**
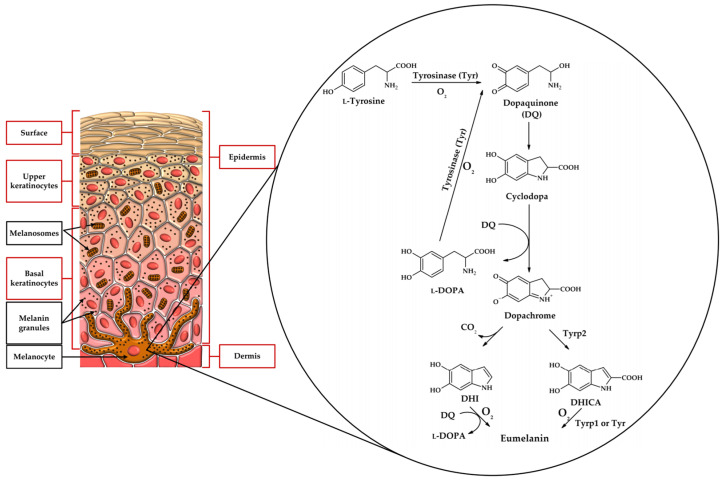
Biosynthetic pathway leading to eumelanin concerning the location of melanocytes in the skin.

Melanin exists in several different forms in nature. The brown-to-black pigment called eumelanin is synthesized when L-tyrosine or L-DOPA is the substrate. Substrates are transformed into melanin using 5,6-dihydroxyindole (DHI) or 5,6-dihydroxyindole-2-carboxylic acid (DHICA) as intermediates. DHICA- and DHI-derived melanins differ from each other. Yellow-to-red pheomelanin can be formed with L-cysteine in the metabolic pathway. This pigment can be found in lips or red hair. Figure 2 presents a simplified pathway leading to the formation of eumelanin. It is worth mentioning that melanogenesis in the skin occurs in specialized cells called melanocytes (Figure 2). Eye colour is also determined by melanins and depends on the amount of the pigment produced in the melanocytes of the iris of the eye.

Melanin formation is a very complex process. In addition to the skin, it can be found in the eyes and the brain, as well as in simpler organisms. The literature mentioned above will introduce the reader to the topic in detail.

Controlling the activity of this enzyme is critically important across several high-value industries. In cosmetics, its inhibition is sought to treat hyperpigmentation disorders, such as freckles, age spots, and melasma [9]. In the food and agriculture sectors, tyrosinase inhibition is crucial for preventing enzymatic browning in fresh produce, thereby extending its shelf life [9]. Furthermore, emerging research suggests that tyrosinase inhibitors may have potential as adjuvant therapies for the treatment of melanoma cancer [10,11].

**Figure 3:**
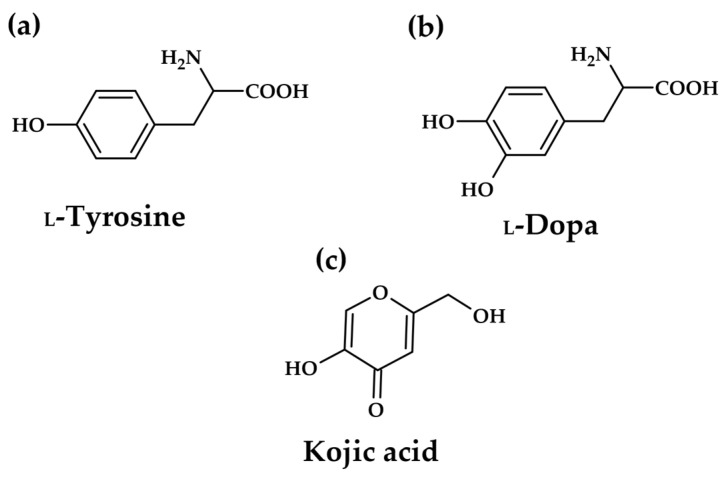
Structures of natural tyrosinase substrates: L-tyrosine (**a**), L-DOPA (**b**), and well-known tyrosinase inhibitor kojic acid–inhibitor research reference, positive control (**c**).

However, the efficacy of existing commercial tyrosinase inhibitors, such as kojic acid (KA, Figure 3c) and arbutin, is often balanced against significant safety and regulatory concerns. Long-term use of these conventional depigmenting agents has been associated with undesirable side effects, including cytotoxicity, skin irritation, and potential DNA damage [12,13]. This intrinsic safety challenge creates a powerful scientific and commercial incentive to discover novel, naturally derived inhibitors that offer comparable or superior potency while possessing a more favourable toxicological profile. The search for alternatives must therefore prioritize compounds demonstrating both low cytotoxicity and defined, beneficial pharmacological mechanisms.

As the key component in melanogenesis, tyrosinase has been receiving considerable attention as an anti-hyperpigmentation target. The above-mentioned kojic acid and arbutin inhibit the enzyme directly, but the definition of tyrosinase inhibitors is sometimes misleading in the literature. In some cases, all compounds interfering with melanin formation are reported as tyrosinase inhibitors, regardless of any enzyme–inhibitor interaction. Most inhibitory investigations are performed using L-tyrosine or _L_-DOPA (Figure 3a,b) as the substrate, and the enzyme activity is evaluated based on dopachrome formation (first colour intermediate in the pathway). The types of inhibitors of melanin biosynthesis were categorized by Chang [14]. Only compounds that bind to the enzyme, reducing its activity, are true enzyme inhibitors.

### 1.2. Tyrosinase Active Site

The mushroom (*Agaricus bisporus*) tyrosinase crystal structure can be found in the Protein Data Bank (PDB, 2Y9X). Visualization of its active site from chain A reveals two copper ions, which are responsible for the enzyme’s catalytic activity, as well as six conserved histidine residues surrounding the metal ions (His62, His85, His94, His259, His263, and His296). Val283 also belongs to the active site as well as the conserved residues of Met280, Glu256, and Asn260 [4].

**Figure 4:**
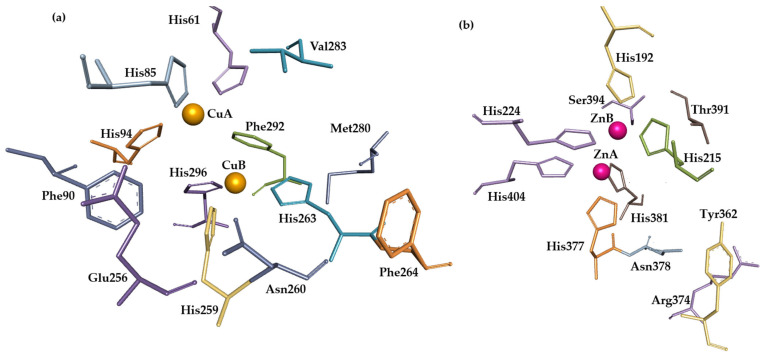
Active sites of *Agaricus bisporus* tyrosinase (**a**) and human tyrosinase-related protein 1 (**b**). Visualization of crystal structures from PDB was created using BIOVIA Discovery Studio Visualizer (Version 2016). Metal ions of Cu and Zn are represented by orange and purple balls, respectively. Other structures are represented by amino acids, depicted as sticks.

There is no crystal structure in human tyrosinase (Trp), but another protein, human tyrosinase-related protein 1 (Trp1), possesses 70% similarity with human tyrosinase, and based on its crystal structure (5M8B), advanced tools like AlphaFold were used to predict the structure of human tyrosinase and perform research, including on the interaction of the enzyme active site with potential inhibitors. Obviously, Trp1 cannot catalyze reactions as tyrosinase does because it possesses two zinc ions in its active site instead of copper ions [15], surrounded by six conserved histidine residues (His192, His224, His404, His215, His377, His381). The geometry of the active site of 2Y9X with ligated tropolone, and analogue zinc-possessing protein, also ligated with tropolone, reveals high similarity. Other amino acid residues found in Trp1 that are considered to be part of the active site are Tyr362, Arg374, Ser394, and Thr391 [16]. Both active sites, from Agaricus bisporus and human tyrosinase-related protein 1, are presented in Figure 4.

### 1.3. Marine Phycochemicals

The marine environment, characterized by extreme ecological pressures, forces its inhabitants to synthesize unique secondary metabolites that provide protection against ultraviolet radiation, predation, and oxidative stress. This rich biochemical diversity positions marine natural products (MNPs) as an essential, yet often underexplored, source of novel chemical scaffolds for pharmacological development [17,18].

Marine algae, encompassing both macroalgae (seaweeds) and microalgae (microscopic, single-celled organisms), represent a vast repository of these bioactive compounds [19,20]. They produce an array of phytochemicals, including polyphenols (such as phlorotannins), carotenoids, polysaccharides, and peptides, which demonstrate significant potential in nutraceutical, pharmaceutical, and cosmeceutical applications [20,21]. The utilization of marine algae offers a pathway toward developing new depigmenting agents that not only address efficacy requirements but also satisfy the growing global demand for natural, safe, and sustainably sourced cosmetic and therapeutic ingredients.

## 2. Algal Sources of Tyrosinase Inhibitors

### 2.1. Macroalgae (Seaweeds)

**Figure 5:**
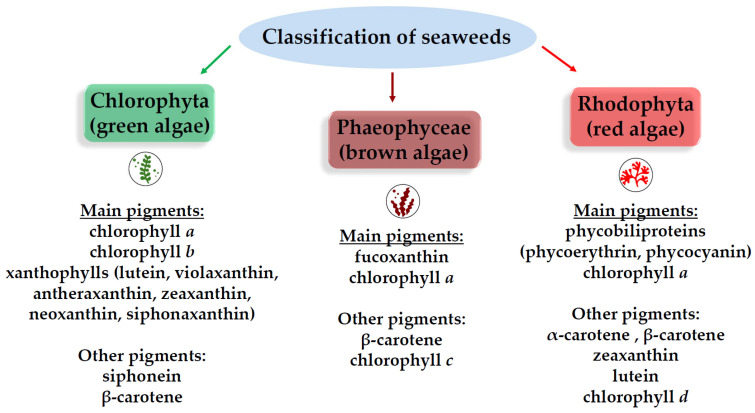
Pigmentation-based groups of seaweeds.

Macroalgae, or seaweeds, are classified into three major groups (brown, red, and green) based on their pigmentation [20] (Figure 5). Brown algae (Phaeophyceae) stand out as the most abundant source of potent tyrosinase inhibitors, largely due to their high concentration of polyphenolic compounds known as phlorotannins.

Brown algae species, including *Ecklonia cava*, *Eisenia bicyclis*, and *Ascophyllum nodosum*, are distinguished by their synthesis of phlorotannins [12,18]. These compounds are complex, highly hydroxylated polyphenols exclusively derived from the monomer phloroglucinol. Phlorotannins exhibit a wide range of molecular sizes, from low-molecular-weight monomers (162 Da) up to large oligomers spanning 650 kDa [21].

Phenolic compounds and polyphenols structurally resemble tyrosinase natural substrates (L-tyrosine and L-DOPA). The hydroxyl group might interact with copper ions in the active site, and the hydrophobic aromatic ring with a highly hydrophobic amino acid active pocket (mostly six histidines (His) surrounding copper ions). All tyrosinases possess a conserved active site [22].

The efficacy of brown algal extracts is notable, as an extract of *Ecklonia cava*—a species recognized for its high total phenolic content (280.11 PGE mg/g)—demonstrated an exceptionally strong anti-tyrosinase effect, registering a half maximal inhibitory concentration (IC50) value of 4.38 ± 0.08 µg/mL [13]. This potency highlights the competitive nature of these natural extracts in comparison to synthetic standards. Related brown seaweeds, such as *Eisenia bicyclis*, show comparable activity with an IC50 of 4.46 ± 0.52 µg/mL [13].

**Figure 6:**
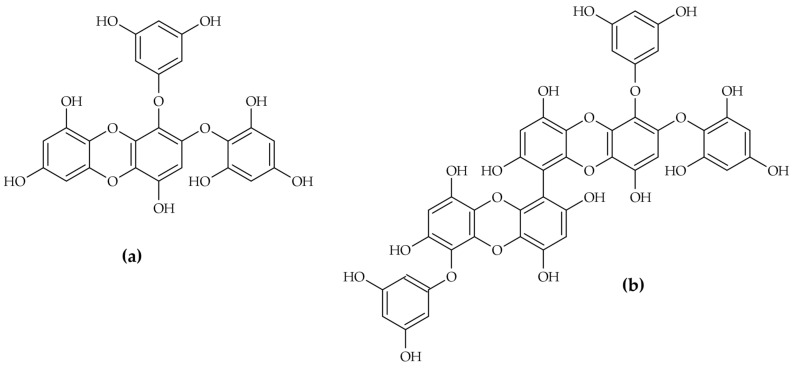
*Ecklonia cava* phlorotannins: 2-phloroeckol (**a**) and 2-O-(2,4,6-trihydroxyphenyl)-6,6′-bieckol (**b**).

The inhibitory activity of phlorotannins is profoundly dependent on their structural complexity, notably the degree of polymerization. Research suggests that larger oligomers exhibit enhanced inhibitory strength, following a general trend of trimer > dimer > monomer [23]. High-purity isolation studies have identified specific phlorotannins that function as potent competitive inhibitors. Two such examples from *E. cava* are 2-phloroeckol and 2-O-(2,4,6-trihydroxyphenyl)-6,6′-bieckol (Figure 6), which yielded impressive IC50 values of 7.0 ± 0.2 µM and 8.8 ± 0.1 µM, respectively [12].

Another important phlorotannin, octaphlorethol A (OPA) derived from *Ishige foliaceae*, inhibits melanin synthesis and tyrosinase activity through a cellular signalling cascade involving the suppression of MITF (microphthalmia-associated transcription factor), tyrosinase, and related proteins via the extracellular signal-regulated kinase (ERK) pathway in B16F10 melanoma cells [24]. This mechanistic diversity confirms that phlorotannins operate not only through direct enzyme binding but also by modulating the upstream genetic and regulatory factors that control melanogenesis.

While brown algae dominate the phenolic inhibitor landscape, red algae (Rhodophyceae) and green algae (Chlorophyceae) contribute to important non-polyphenolic agents. Red algae such as *Sargassum latiuscula* yield bromophenols, where an increased number of bromine groups correlates positively with potent mushroom tyrosinase inhibitory activity [23]. This suggests halogenation is a viable strategy for enhancing the depigmenting potential of algal compounds.

Furthermore, lipophilic extracts from red algae have demonstrated significant efficacy. Among tested species, including *Sargassum polycystum* and *Caulerpa lentillifera*, the lipophilic extracts of *Gracilaria fisheri* exhibited the strongest anti-tyrosinase activity [20]. Detailed molecular analysis identified stigmasterol as a key bioactive constituent. This sterol showed low toxicity of in vitro cytotoxicity assays (IC50 = 3.38 ± 0.28 µg/mL) and had no adverse effects at concentrations of 0.006 µg/mL [13]. ADMET (absorption, distribution, metabolism, excretion, and toxicity prediction) highlighted stigmasterol’s favourable skin permeability and selectivity, with no significant off-target interactions against serine proteases, such as trypsin and chymotrypsin [18].

When evaluating natural product candidates, commercial adoption is driven by more than sheer potency; it necessitates a strong safety profile and acceptable pharmacokinetics. Compounds like stigmasterol, which have been rigorously assessed for favourable ADMET characteristics, represent superior R&D investments, providing a clear path toward safe and selective topical formulation, even if their in vitro potency might be marginally lower than that of synthetic analogues. Molecular modelling strategies are used to identify patterns in ADMET data and convert them into knowledge (predictions), but predictions can not be considered as straightforward molecular modelling results. Nowadays, traditional chemometrics is evolving into advanced machine learning methods [25].

Table 1 synthesizes the quantitative performance of key algal inhibitors, demonstrating their competitive position relative to synthetic standards like kojic acid (KA).

### 2.2. Microalgae and Cyanobacteria

Microalgae and cyanobacteria (often categorized together due to historical context and application overlapping) are increasingly recognized as superior sources for highly specific, high-value compounds. Their cultivation under controlled bioreactor conditions enables scalable and predictable biomass production, making them ideal for pharmaceutical and high-end cosmeceutical development.

**Figure 7:**
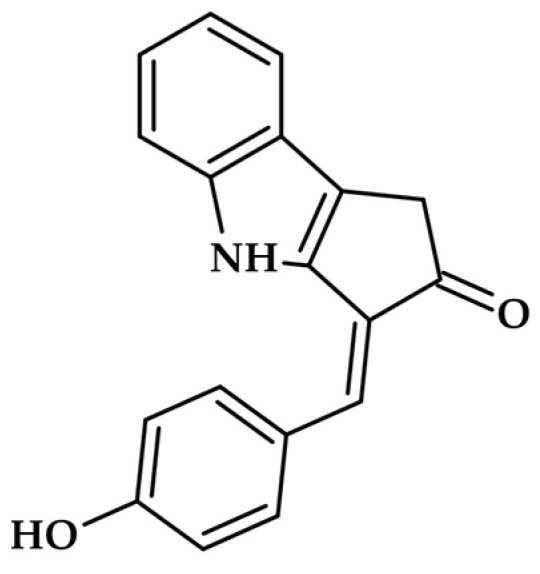
Scytonemin monomer.

The carotenoid class of pigments, which includes apocarotenoids, represents a potent group of microalgal inhibitors. The most striking example discovered recently is scytonemin monomer (ScyM—Figure 7), a precursor to the cyanobacterial sunscreen pigment scytonemin.

ScyM exhibits exceptional potency against mushroom tyrosinase, achieving an IC50 of 4.90 µM [10]. Critically, this activity is significantly stronger than the commercial standard, kojic acid, establishing ScyM as a promising lead compound for skin whitening and potentially as an adjuvant therapy for melanoma [10]. 

Other microalgae, such as *Dunaliella* species, biosynthesise rich concentrations of carotenoid pigments, including β- and α-carotene, zeaxanthin, and lutein [11]. Mechanistic studies on related apocarotenoids, such as bixin and norbixin, suggest an allosteric mode of inhibition, where they bind to the tyrosinase enzyme through hydrophobic interactions at sites distinct from the primary active pocket [26]. Furthermore, astaxanthin (ATX), another prominent microalgal carotenoid, has demonstrated chemopreventive effects against UV-induced skin tumorigenesis by inhibiting tyrosinase activity and modulating oxidative stress, underscoring the dual-action benefit of these pigments [27].

Structurally, carotenoids like scytonemin differ significantly from classical phenolic tyrosinase inhibitors such as kojic acid or phlorotannins. While phenolic inhibitors typically feature multiple hydroxyl groups that can chelate the copper ions in tyrosinase’s active site or compete with natural substrates (L-tyrosine, L-DOPA), carotenoids possess extended conjugated polyene chains with cyclohexene or aromatic rings at their termini [28]. Scytonemin’s unique alkaloid structure contains a fused indole–phenolic moiety that provides both aromatic character and potential for copper coordination [29]. This structural feature enables it to interact with the enzyme’s active site in a manner distinct from simple phenols. In contrast to the relatively small, flexible molecules like kojic acid (MW 142 Da), scytonemin monomer (MW ~544 Da) presents a larger, more rigid scaffold with multiple potential binding sites. This structural complexity may contribute to its superior inhibitory potency (IC50 = 4.90 µM vs. kojic acid IC50 = 15.6 µM) and slow-binding kinetics. The comparison reveals that effective tyrosinase inhibitors need not strictly mimic the substrate structure but can achieve inhibition through diverse molecular architectures capable of disrupting the catalytic mechanism or blocking substrate access to the binuclear copper centre [12].

This distinction in approach is fundamental for R&D strategy: while macroalgae provide potent, complex extracts, microalgae deliver specific, chemically novel small molecules, such as ScyM. These purified compounds are highly desirable for targeted pharmaceutical or cosmeceutical formulations where dosage precision and predictable pharmacokinetics are paramount.

Microalgae, particularly cyanobacteria like Spirulina (*Arthrospira platensis*), are substantial sources of protein-based inhibitors. C-phycocyanin (PC), the characteristic blue pigment of *A. platensis*, is a phycobiliprotein with known anti-melanogenic activity. In cellular models (SK-Mel-28 melanoma cells), phycocyanin demonstrated tyrosinase inhibition potential after 60 min/120 min–30.88 µg/mL/39.87 µg/mL, respectively, a result comparable to the ascorbic acid control (34.06 µg/mL/17.48 µg/mL) [30].

Despite this potency, formulating protein-based compounds like PC presents unique challenges related to stability and skin penetration. Successful commercialisation requires overcoming these obstacles through advanced delivery systems. A study demonstrated the feasibility of encapsulating *A. platensis* extracts containing phycocyanin into Nanostructured Lipid Carriers (NLCs) [21]. This NLC gel preparation was physically and chemically stable and maintained robust inhibitory activity, proving that lipid-based encapsulation can effectively stabilize and deliver fragile microalgal bioactives for topical application [21].

Microalgae are also rich in other bioactive peptides [18]. Given the strong performance of phycocyanin and the known efficacy of peptides in enzyme inhibition, further investigation into short, stable tyrosinase inhibitory peptides (TIPS) derived from microalgal protein hydrolysates represents a major avenue for future research and product development [11].

In addition to pigments and proteins, certain microalgae synthesize low-molecular-weight phenolic compounds. *Picochlorum* sp. SBL2, for instance, possesses a high total phenolic content, with salicylic, coumaric, and gallic acids identified as major components [11]. These phenolics, often coexisting with carotenoids, contribute synergistically to the observed anti-tyrosinase and antioxidant properties of the extracts [11]. Extracts from *Nitzschia* sp. microalgae are also documented in patents for their use in inhibiting tyrosinase activity and melanin formation [11].

However, the efficacy of microalgal extracts can vary significantly depending on the strain, cultivation conditions, and extraction solvent. For example, a methanol extract of *Scenedesmus dimorphus* showed an IC50 value (1799 mg/mL) significantly higher than the kojic acid positive control (0.002 mg/mL), indicating a need for careful strain selection and optimized extraction protocols to ensure commercially relevant potency [31].

## 3. Biochemical Mechanisms and Enzyme Kinetics of Algal Inhibitors

A nuanced understanding of enzyme kinetics is crucial for rational drug design and predicting in vivo efficacy. Algal inhibitors exhibit diverse mechanistic profiles, binding to tyrosinase through competitive, non-competitive, or allosteric modes.

Inhibitors are classified based on their interaction site relative to the active enzyme (E) and the enzyme–substrate complex (E-S) [32,33]:

1. Competitive inhibitors: These compounds bind reversibly to the free enzyme at the active site, directly blocking the substrate (L-tyrosine or L-DOPA) [29]. Purified phlorotannins, such as 2-phloroeckol and 2-O-(2,4,6-trihydroxyphenyl)-6,6′-bieckol, typically fall into this category [12].
2. Non-competitive inhibitors: These compounds bind to the enzyme at an allosteric site distinct from the active centre. They interact with both the free enzyme and the E-S complex with the same equilibrium constant [34]. Interestingly, crude extracts of brown algae like *Ecklonia cava* and *Eisenia bicyclis* often exhibit non-competitive inhibition kinetics [13].
3. Allosteric inhibitors: Some enzymes possess more than one site able to bind ligands. A ligand that binds at one site induces structural changes in the protein that are transmitted via the polypeptide chain to the other active site, diminishing the binding ability of the substrate to its active site, is called an allosteric inhibitor [34]. An example of such an inhibitor is phlorotannins, sourced from *Ecklonia stolonifera* phlorofucofuroeckol-A [35]. Carotenoids, such as apocarotenoids (e.g., bixin), are also reported to inhibit tyrosinase allosterically through hydrophobic interactions [29].
4. Mixed-type inhibitors: These compounds bind to both the free enzyme and the E-S complex but with different affinities [29,30]. An algal example of a tyrosinase mixed-type inhibitor is a scytonemin monomer [10].

While the scytonemin monomer (ScyM) demonstrates exceptional potency against mushroom tyrosinase (IC50 = 4.90 µM) and shows superior performance compared to kojic acid [12], several critical limitations temper this promising profile and must be addressed before clinical translation. First, most tyrosinase inhibitor research, including studies on ScyM, relies on mushroom (*Agaricus bisporus*) tyrosinase rather than human enzymes due to commercial availability and cost considerations. However, significant structural differences exist between fungal and mammalian tyrosinases, particularly in the geometry and accessibility of the substrate binding pocket, which can dramatically affect inhibitor efficacy [36,37]. Studies have shown that compounds like hydroquinone and kojic acid exhibit markedly different inhibitory potencies against mushroom versus human tyrosinase, with IC50 values differing by orders of magnitude [38]. Without validation against human tyrosinase or in human melanocyte models, the translational potential of ScyM remains uncertain. Second, delivery presents a formidable challenge: the molecular weight of ScyM (~544 Da) exceeds the optimal range for passive skin penetration (typically <500 Da according to Lipinski’s rule), and its lipophilic character may limit aqueous solubility in cosmetic formulations. Encapsulation strategies using nanoparticles, liposomes, or cyclodextrin complexes may be necessary, but they also add complexity and increase manufacturing costs. Third, comprehensive toxicity data for purified ScyM remain limited. While cyanobacterial biomass containing scytonemin has traditional use, purified ScyM has not undergone rigorous dermatological safety testing, chronic exposure studies, genotoxicity assessment, or photostability evaluation—critical requirements for cosmetic applications. Finally, commercial scalability faces substantial obstacles: cyanobacteria grow more slowly compared to other microalgae (doubling times of 3–7 days vs. 1–2 days for Chlorella), and scytonemin biosynthesis requires UV stress induction, complicating controlled production and increasing costs. These challenges highlight the substantial development pathway required to transform a potent laboratory candidate into a commercially viable, safe, and effective cosmetic active ingredient.

The distinction between the crude extract (non-competitive) and the purified small oligomers (competitive) from *E. cava* suggests a major difference in mechanisms. The non-competitive behaviour of the crude extract is likely mediated by high-molecular-weight compounds, which may physically alter the enzyme’s conformation or act as general protein binders outside the active site [11,13]. Conversely, the targeted inhibition by purified, smaller oligomers demonstrates specific binding to the active site.

A particularly advantageous kinetic profile observed in high-value phlorotannins is slow-binding inhibition, where the inhibitory activity strengthens over time upon preincubation with the enzyme [12]. This mechanism suggests a prolonged and sustained therapeutic effect, a highly desirable trait for chronic conditions like hyperpigmentation.

Analysis of the kinetics revealed two distinct slow-binding mechanisms among the potent phlorotannins isolated from *Ecklonia cava* [12]:

1. 2-phloroeckol: Single-step mechanism (mechanism A). This molecule exhibits a competitive, slow-binding profile characterized by a single-step association, where the ligand slowly binds directly to the active site to form a stable encounter complex [12].
2. 2-O-(2,4,6-trihydroxyphenyl)-6,6′-bieckol: Two-step mechanism (mechanism B). This compound, which has a higher molecular weight and more complex structure, follows a more complex mechanism. It involves an initial rapid interaction followed by a slower enzyme isomerisation that results in a new, long-lived conformational state of the enzyme [12]. This ability to induce a structural shift and persistently inactivate the enzyme imparts a significant pharmacological advantage.

The comparison of these two competitive inhibitors demonstrates that the structural complexity, particularly the molecular size and the greater number of hydroxyl groups in complex polyphenols like 2-O-(2,4,6-trihydroxyphenyl)-6,6′-bieckol [11], dictates the binding mechanism. Distinguishing the mechanism allows, therefore, rational design or modification of inhibitor candidates to favour the two-step mechanism, maximizing the longevity and effectiveness of the depigmenting agent. The mechanistic classification of key algal tyrosinase inhibitors is shown in Table 2. 

Molecular docking simulations also provide crucial insights into the binding interactions that drive high inhibitory potency. For 2-phloroeckol and 2-O-(2,4,6-trihydroxyphenyl)-6,6′-bieckol, the simulations confirmed their role as competitive inhibitors, physically covering the area around the active site of tyrosinase [12].

Both potent inhibitors interact directly with core catalytic residues, notably His85 and Asn260 [12]. 2-phloroeckol forms six hydrogen bonds with three amino acids (Glu256, Asn260, and Met280) and exhibits a π-π bond interaction with His85 [12]. 2-O-(2,4,6-trihydroxyphenyl)-6,6′-bieckol, due to its larger size, forms extensive hydrogen bonding networks, utilizing seven hydroxyl groups to interact with eight amino acids, including Lys79, Asn81, Cys83, and His85 [12]. The AutoDock scores calculated using AutoDock Version 4.2 (along with AutoDockTools) mirrored their high in vitro IC50 values, reinforcing the structural basis of their strong binding affinity [12].

A significant obstacle in cross-study comparative evaluation of algal tyrosinase inhibitors is the inconsistency in reported IC50 values [11]. The potency metrics are highly sensitive to varied assay conditions, including differences in substrate concentration, enzyme source (e.g., batch variability of mushroom tyrosinase), and incubation time [11].

To enable practical and reliable comparison for R&D purposes, studies must move beyond isolated reporting toward standardized metrics. The critical path forward is the consistent application of Relative Inhibitory Activity (RA), which involves running a well-known standard inhibitor, such as kojic acid, simultaneously under identical assay conditions and normalizing the results [11]. Adoption of standardized protocols would ensure that potency comparisons are pharmacologically meaningful and can reliably guide preclinical decision-making.

## 4. Sustainable Extraction and Green Chemistry

Scaling up the production of algal tyrosinase inhibitors requires moving from laboratory-scale organic solvent extraction to green, efficient, and cost-effective commercial processes. The fragility of high-value compounds, such as phlorotannins and phycobiliproteins, necessitates innovative extraction and formulation technologies. Traditional extraction methods for polyphenols often involve large volumes of conventional organic solvents, which present environmental hazards and increase process costs [18]. Modern industrial practices are rapidly shifting toward green chemistry solutions.

Phlorotannins are moderately hydrophilic polyphenolic compounds with molecular weights ranging from 126 Da (phloroglucinol monomer) to over 650 kDa for highly polymerized structures [39]. Their solubility characteristics present both opportunities and challenges for extraction and characterization. Unlike terrestrial tannins, phlorotannins exhibit polar character due to their multiple hydroxyl groups, making them soluble in polar and semi-polar solvents [40]. The most commonly employed extraction solvents are aqueous mixtures of ethanol (30–80% *v*/*v*), methanol (30–70% *v*/*v*), and acetone (40–70% *v*/*v*) [41,42]. These binary solvent systems exploit synergistic effects: water provides polarity for hydrogen bonding with hydroxyl groups, while organic solvents reduce surface tension and facilitate penetration into algal cell matrices [38].

Optimization studies have demonstrated that extraction efficiency is highly dependent on solvent composition. For *Fucus vesiculosus*, maximum phlorotannin recovery (1.77 mg phloroglucinol equivalents/g dry seaweed) was achieved with 50–70% acetone [40]. Similarly, *Sargassum fusiforme* extractions yielded optimal results with 30% ethanol at 25 °C for 30 min [38]. The polarity of the solvent system directly influences which molecular weight fractions are preferentially extracted: lower ethanol concentrations (30–50%) favour extraction of higher-molecular-weight, more polar phlorotannins, while higher concentrations (70–80%) extract lower-molecular-weight oligomers [41]. Temperature is a critical parameter requiring careful balance—elevated temperatures (40–60 °C) increase extraction kinetics but risk oxidative degradation of phlorotannins, which are susceptible to autoxidation and polymerization reactions that can alter their chemical structures and reduce bioactivity [38].

Green extraction technologies have emerged as sustainable alternatives to conventional methods. Natural Deep Eutectic Solvents (NADESs), typically composed of choline chloride paired with hydrogen bond donors such as lactic acid or glycerol, have demonstrated extraction efficiencies comparable to those of traditional organic solvents while offering a reduced environmental impact [42]. Aqueous NADES solutions based on choline chloride and lactic acid extracted phlorotannins from *Fucus vesiculosus* and *Ascophyllum nodosum* with efficiencies matching those of acetone and ethanol, and nearly 10-fold higher than pure NADES [42]. Ultrasound-assisted extraction (UAE) and microwave-assisted extraction (MAE) represent additional sustainable approaches that reduce solvent consumption and extraction time while maintaining or improving yields [43,44]. Supercritical fluid extraction using CO2 with ethanol or water as co-solvents has demonstrated selective recovery of phlorotannins with minimal thermal degradation [45].

Following extraction, crude extracts contain not only phlorotannins but also interfering substances including polysaccharides (alginates, fucoidans, laminaran), proteins, pigments (fucoxanthin, chlorophylls), and mannitol—a polyol that can constitute 2–10% of dry algal biomass [41]. Purification is therefore essential for structural characterization and bioactivity assessment. The most widely employed purification strategy involves liquid–liquid partitioning, where aqueous crude extracts are sequentially fractionated with organic solvents of increasing polarity: n-hexane (for lipid removal), ethyl acetate (for phlorotannin enrichment), and n-butanol (for polar phenolics) [28,40]. This approach achieved phlorotannin purities of 88.48 mg phloroglucinol equivalents per 100 mg extract for *Sargassum fusiforme* [38].

Solid-phase extraction (SPE) using C18-functionalized silica provides an alternative approach that simultaneously removes interfering carbohydrates while concentrating phlorotannins [41]. More sophisticated purification employs macroporous adsorption resins, particularly Diaion HP-20, which selectively adsorbs phlorotannins via hydrophobic interactions and allows desorption with aqueous ethanol [44]. This method achieved 2.4-fold enrichment with simultaneous 100% removal of mannitol for *Durvillaea incurvata* extracts [44]. Size exclusion chromatography (SEC) offers molecular-weight-based fractionation, enabling separation of oligomeric phlorotannins by their degree of polymerization [46]. For the isolation of individual phlorotannins, preparative-scale techniques, including centrifugal partition chromatography (CPC) and two-dimensional liquid chromatography (2D-LC), have been successfully implemented [47].

Structural characterization of phlorotannins does not require chemical degradation or derivatization when employing modern analytical platforms. Ultra-High-Performance Liquid Chromatography coupled with high-resolution tandem mass spectrometry (UHPLC-HRMS/MS) enables the direct analysis of intact phlorotannin oligomers and polymers [41,44]. Negative electrospray ionization (ESI-) generates [M-H]- molecular ions, and collision-induced dissociation provides fragmentation patterns revealing phloroglucinol unit connectivity and linkage types (C-C bonds, ether linkages, or dibenzodioxin bridges) [47]. Nuclear Magnetic Resonance (NMR) spectroscopy, particularly 1H-NMR and 13C-NMR with 2D techniques (COSY, HSQC, HMBC), provides unambiguous structural elucidation of purified phlorotannins without requiring chemical modification [18]. Matrix-Assisted Laser Desorption/Ionization Time-of-Flight Mass Spectrometry (MALDI-TOF-MS) facilitates analysis of high-molecular-weight phlorotannins (>1000 Da) that are challenging to ionize by ESI [48]. Infrared spectroscopy (FTIR) rapidly confirms the presence of characteristic phlorotannin functional groups—broad O-H stretching (3200–3600 cm^−1^), aromatic C=C vibrations (1400–1600 cm^−1^), and C-O-C linkages (1000–1200 cm^−1^)—without sample destruction [49].

Accelerated solvent extraction (ASE) and pressurized liquid extraction (PLE) are innovative techniques that are highly effective in optimizing extraction yields and are essential for preventing the degradation of sensitive bioactive structures, thereby maintaining the high bioactivity observed in crude samples [50].

NADESs (Natural Deep Eutectic Solvents) represent a major advancement in eco-responsible extraction. These solvents, typically composed of biodegradable components such as choline chloride and lactic acid, are environmentally friendly and demonstrate exceptional efficiency [51]. Aqueous NADES solutions, such as lactic acid/choline chloride, are suitable for the simultaneous extraction of both hydrophilic compounds (phlorotannins, ascorbic acid) and lipophilic compounds (fucoxanthin) from brown algae like *Fucus vesiculosus* [51]. Crucially, the use of NADESs not only improves yield and environmental impact but also enhances the stability of the active metabolites extracted, minimizing degradation and reducing the cost of goods (COG) for manufacturers [51]. The successful application of optimized green extraction methods is evidenced by specific fractions achieving inhibition rates (e.g., 84.5 ± 1.53% for an LLE_FAE fraction) that closely rival the positive control kojic acid (88.96 ± 1.05%) [50].

Advanced chromatographic and spectroscopic techniques are indispensable for accurate identification and quantification of tyrosinase inhibitors from algal sources. For phlorotannins, methods include spectrophotometry, Nuclear Magnetic Resonance (NMR) spectroscopy, and mass spectrometry (MS) [52]. High-Performance Liquid Chromatography (HPLC), coupled with various detectors (UV-Vis, fluorescence, or MS), remains the gold standard for separating and quantifying individual compounds in complex extracts [38,53]. For microalgal pigments, such as carotenoids and phycocyanin, UV-Vis spectrophotometry provides rapid quantification, while LC-MS/MS offers superior specificity for structural elucidation [54]. Ultra-High-Performance Liquid Chromatography (UHPLC) has accelerated analysis times while improving resolution, making it particularly valuable for routine quality control in industrial settings [55]. Two-dimensional liquid chromatography coupled with mass spectrometry has enabled the identification of up to 50 phlorotannin compounds from brown seaweed extracts [47]. The integration of these analytical platforms ensures comprehensive characterization of algal extracts, supporting both research development and regulatory compliance in commercial applications.

Many potent algal compounds suffer from poor stability and limited skin permeability. Overcoming these challenges requires sophisticated formulation strategies. The successful integration of *Spirulina platensis* extracts into Nanostructured Lipid Carriers (NLCs) provides a case study [23]. This encapsulation technique produced a physically and chemically stable gel that retained its tyrosinase inhibitory activity. NLC technology enhances bioavailability and provides improved protection for fragile compounds, such as phycocyanin, thereby transforming highly active but unstable bioactives into viable commercial products [23]. Phlorotannins, despite their high tyrosinase-inhibitory activity, are prone to oxidation and polymerisation under exposure to light, oxygen, and alkaline pH, necessitating stabilization approaches [38]. Encapsulation in cyclodextrins has proven effective: β-cyclodextrin and hydroxypropyl-β-cyclodextrin inclusion complexes improved the stability of *Ecklonia cava* extracts by protecting active compounds from degradation while enhancing aqueous solubility [39]. Nanoencapsulation technologies offer superior delivery performance: solid lipid nanoparticles (SLNs) and Nanostructured Lipid Carriers (NLCs) loaded with phycocyanin achieved sustained release profiles and demonstrated enhanced skin penetration in ex vivo studies using Franz diffusion cells, with 2.5-fold greater permeation compared to free pigment [56]. Liposomal formulations have similarly improved the bioavailability of lipophilic carotenoids from *Dunaliella*, overcoming their inherent poor water solubility. For industrial-scale applications, emulsion-based systems provide practical solutions: oil-in-water emulsions stabilized with algal polysaccharides (alginates from brown algae, carrageenans from red algae) not only deliver lipophilic inhibitors but also provide additional skin-conditioning and moisturizing benefits [57]. Hydrogel matrices incorporating alginate or carrageenan have been successfully used to formulate Spirulina extracts for topical application, combining controlled release kinetics with bioadhesive properties that extend skin contact time and improve active ingredient penetration [58]. Nanoemulsions (droplet size < 200 nm) represent another promising approach, offering enhanced stability, improved skin penetration, and aesthetic appeal compared to conventional emulsions. These formulation innovations bridge the gap between the potency of raw materials measured in vitro and the performance of consumer products in clinical applications, ensuring that the inherent biological activity of algal inhibitors translates into measurable efficacy in skin-lightening applications. The strategic selection of delivery systems must balance multiple factors: enhancement of bioavailability, protection from degradation, manufacturing feasibility, cost-effectiveness, regulatory compliance, and consumer acceptability—all critical determinants of successful market introduction.

## 5. Commercial Application Landscape and Future Biorefineries

The market positioning of algal tyrosinase inhibitors is strong, driven by consumer preference for natural ingredients and the need for multi-functional cosmetic agents.

Despite their promising in vitro bioactivities, the practical application of phlorotannins is significantly limited by their low bioavailability following oral administration. Bioavailability—defined as the fraction of an administered compound that reaches systemic circulation in its active form—is determined by factors including gastrointestinal stability, intestinal absorption, first-pass metabolism, and systemic clearance [59]. Phlorotannins face multiple bioavailability challenges that differ substantially from their terrestrial polyphenol counterparts.

High-molecular-weight phlorotannins (>1000 Da) exhibit poor absorption in the small intestine due to their size and hydrophilicity, which prevent passive diffusion across intestinal epithelial membranes [58,59]. Human intervention studies have demonstrated that only approximately 14% of phlorotannins from *Fucus vesiculosus* are absorbed in the upper gastrointestinal tract [60]. Following oral administration of phlorotannin-rich capsules (400 mg containing 101.89 mg polyphenols), plasma and urine analysis revealed that the majority of metabolites appeared at late time points (6–24 h post-ingestion), indicating limited small intestinal absorption followed by extensive colonic metabolism [59]. High-molecular-weight phlorotannins undergo bacterial fermentation in the large intestine, producing lower-molecular-weight metabolites, including hydroxytrifuhalol A, 7-hydroxyeckol, and C-O-C dimers of phloroglucinol, which are subsequently absorbed and excreted in the urine [61].

Phlorotannins are chemically unstable under the physiological conditions of the gastrointestinal tract. They undergo rapid transformation in the acidic environment of the stomach (pH 1–3) and the alkaline conditions of the small intestine (pH 6.5–7.5), with extensive metabolic processing by digestive enzymes [61]. Phase II metabolism—glucuronidation and sulfation catalyzed by UDP–glucuronosyltransferases and sulfotransferases in the intestinal epithelium and liver—produces conjugated metabolites with altered biological activities [59]. These conjugates exhibit reduced antioxidant capacity compared to parent compounds and are rapidly cleared via renal excretion [62]. Additionally, phlorotannins can form complexes with dietary proteins, minerals (iron, calcium), and other polyphenols, further reducing their bioavailability [63].

Substantial inter-individual variation in phlorotannin metabolism has been observed, attributed to differences in gut microbiota composition, expression levels of metabolizing enzymes (cytochrome P450 isoforms, phase II conjugation enzymes), and intestinal transit times [59]. In clinical studies, only 15 out of 24 volunteers showed detectable phlorotannin metabolites in plasma and urine, highlighting this variability [59].

To address these limitations in bioavailability, advanced delivery systems have been investigated. Nanoencapsulation technologies—including liposomes, solid lipid nanoparticles (SLNs), Nanostructured Lipid Carriers (NLCs), polymeric nanoparticles, and nanoemulsions [64]—protect phlorotannins from degradation in the gastrointestinal tract while enhancing absorption through increased surface area and potential for receptor-mediated uptake [61]. Cyclodextrin inclusion complexes improve the aqueous solubility of phlorotannins and provide protection against oxidation [59]. Hydrogel matrices fabricated from alginate, chitosan, or carrageenan enable sustained release and mucoadhesion, prolonging intestinal residence time and improving absorption kinetics [62]. These formulation strategies represent critical enabling technologies for translating the potent in vitro activities of phlorotannins into clinically meaningful in vivo efficacy. Future research must prioritize comprehensive pharmacokinetic studies in human subjects to definitively establish bioavailable doses and optimal delivery formats for therapeutic applications.

### 5.1. Cosmeceutical and Skin-Whitening Applications

The increasing global demand for sustainable and natural ingredients has accelerated the integration of seaweed-derived extracts into the cosmetics sector [17,65]. Algal compounds, particularly phlorotannins, are highly prized because they are multi-functional. They display potent anti-melanogenic activity alongside synergistic properties, including anti-aging, antioxidant, anti-inflammatory, and photoprotective effects [21]. For instance, dieckol, isolated from *E. cava*, has demonstrated a strong photoprotective effect, effectively scavenging intracellular reactive oxygen species (ROS) and mitigating damage induced by UVB radiation, positioning it as a powerful ingredient for comprehensive skin defense [21].

The commercial viability of these extracts is already established, with companies actively developing and patenting skin-whitening compounds derived from marine algae [66]. Extracts from green algae, such as *Chlamydomonas reinhardtii*, are utilized in commercial products for benefits encompassing cellular renewal, hydration, and anti-inflammation, showcasing the breadth of application for microalgal sources [67].

Several companies have established commercial positions in the marine-derived skin-whitening market. French company Codif Technologie Naturelle produces “Algisium C^®^,” a standardized extract from Laminaria digitata marketed as a skin brightening ingredient with documented tyrosinase inhibitory activity. Korean biotechnology firms have been particularly active in this space, with companies patenting formulations that incorporate *Ecklonia cava* phlorotannin extracts, demonstrating both in vitro and in vivo efficacy in reducing melanin content [12]. In Japan, companies have developed commercial-scale production systems for astaxanthin from *Haematococcus pluvialis*, which, though primarily marketed for dietary supplements, has secondary applications in anti-pigmentation cosmetics due to its antioxidant properties that indirectly affect melanin formation [68] (Shah et al., 2016). European skincare brands have incorporated *Chlorella vulgaris* extracts as multi-functional ingredients offering both antioxidant and skin-brightening properties. Major multinational cosmetics corporations, including L’Oréal, Beiersdorf, and Shiseido, have invested in marine biotechnology research, with several patent applications covering algal-derived depigmenting agents [69]. The successful market presence of these products demonstrates established supply chains, quality control protocols, and regulatory compliance frameworks necessary for commercial viability. The Asia-Pacific region, particularly Japan, South Korea, and China, represents the largest market for these products, accounting for over 50% of global skin-lightening product sales [69].

### 5.2. Medical and Agricultural Potential

Beyond cosmetics, highly potent compounds like scytonemin monomer (ScyM), which showed superiority over kojic acid, are being investigated for potential roles in the medical sector, specifically as adjuvant therapeutic agents for melanoma cancer [10]. Furthermore, the broad utility of tyrosinase inhibitors extends to the food industry, where they play a critical role as anti-browning agents in preserving the aesthetic and commercial value of perishable produce [9].

In the food industry, tyrosinase inhibitors from algal sources show promise as natural anti-browning agents for fruits and vegetables. Sulfite compounds have traditionally been used but face regulatory restrictions due to health concerns. Natural alternatives under investigation include polyphenolic extracts from brown seaweeds, which can prevent enzymatic browning in apple slices, potato products, and mushrooms [70]. Phlorotannin-rich extracts from *Ascophyllum nodosum* and *Fucus vesiculosus* have demonstrated the ability to delay oxidation in many food products [71]. Ascorbic acid (vitamin C) is currently the most widely used natural anti-browning agent, acting as a reducing agent that converts quinones back to phenols, but its efficacy is limited by rapid oxidation. Algal extracts offer the advantage of dual-action mechanisms: direct tyrosinase inhibition combined with antioxidant activity, providing more sustained protection against enzymatic browning compared to single-mechanism compounds [72]. Carrageenan-based edible films incorporating seaweed extracts have shown effectiveness in preserving the visual quality of minimally processed lettuce and fresh-cut vegetables [73].

### 5.3. Comparative Sustainability Assessment (LCA)

Sustainability is a cornerstone of modern natural product development. Life Cycle Analysis (LCA) is a strategic tool essential for evaluating the environmental impact and economic viability of algal bioprocessing [19,74].

Microalgae offer distinct advantages in sustainability and industrial control. They boast rapid growth rates, highly efficient carbon dioxide utilization, and the flexibility to be cultivated in controlled bioreactors using diverse water sources (including wastewater) [75]. This controlled environment ensures batch-to-batch consistency in bioactive compound composition, mitigating the high variability often associated with wild-harvested macroalgae, which are influenced by seasonal and environmental fluctuations [74]. The ability to accurately assess the environmental and economic metrics via LCA promotes sustainable practices and lowers the production risk for manufacturers requiring standardized inputs for Good Manufacturing Practice (GMP) standards (Table 3) [19,75].

Macroalgae sourcing often relies on wild harvesting or nascent aquaculture [76]. While macroalgae are abundant, the reliance on field conditions can lead to unpredictable yields of desired phytochemicals [74]. Ultimately, the economic feasibility of microalgal production hinges on maximizing the extraction of high-value components (like ScyM or phycocyanin) to offset the typically high capital and operating costs of photobioreactors.

### 5.4. Algal Safety

Algae food safety (AFS) is still a nascent requirement that should be considered by policy-makers, researchers, producers, and consumers. Algae provide multiple valuable compounds that can be used in multiple fields, but at different stages of development, there are hazardous processes related to AFS, including contamination, e.g., biotoxins, radioactive compounds, or heavy metals. The relevant stages include cultivation, harvesting, drying, packaging, and storage. Quality assessment regarding toxicology and microbiological standard analysis has been introduced. However, gaps are still present with regard to safety. Producers must implement GMP rules, and researchers need to focus on developing new, efficient analytical methods to detect contamination. Simultaneously, consumers have to be aware of the potential hazards [77,78]. 

A comprehensive safety evaluation is paramount before the commercial implementation of any tyrosinase inhibitor. Established inhibitors like kojic acid face significant limitations: cytotoxicity concerns, skin irritation, and potential sensitization have led to regulatory restrictions in several countries, with the Cosmetic Ingredient Review concluding that safe use is limited to concentrations below 1% [79,80]. Hydroquinone, despite its efficacy, is banned in cosmetics within the European Union due to cytotoxicity, carcinogenic concerns, and association with ochronosis [81]. Hydroquinone causes DNA damage leading to melanocyte death, and chronic exposure has been linked to thyroid disorders and liver damage [81]. In contrast, preliminary safety data for algal-derived inhibitors appear promising. Phlorotannins from brown seaweeds have shown minimal cytotoxicity in multiple cell line studies (HaCaT keratinocytes, B16F10 melanoma cells) at concentrations well above their effective inhibitory doses [12]. For instance, 2-phloroeckol exhibited no significant cytotoxicity at concentrations up to 100 µM, while its IC50 for tyrosinase inhibition was only 0.61 µM—providing a favourable therapeutic window exceeding 160-fold. Phycocyanin from Spirulina is Generally Recognized As Safe (GRAS) by the FDA and has been consumed as a dietary supplement for decades without adverse effects, supporting its safety profile for topical applications [56]. However, critical gaps remain, as most published studies focus on acute cytotoxicity rather than chronic exposure, dermal sensitization potential, phototoxicity, or photocarcinogenicity. Future development must include comprehensive dermatological safety testing, including repeated insult patch tests (RIPT), human maximization tests, and long-term exposure studies to establish definitive safety profiles comparable to pharmaceutical standards.

## 6. Conclusions and Critical Outlook

Marine algae, both macroalgae and microalgae, represent a crucial and largely unexploited resource for the discovery and development of potent, safe, and sustainable tyrosinase inhibitors. The scientific literature confirms that several algal compounds and extracts possess IC50 values that are highly competitive with, and in some cases superior to, established synthetic standards, such as kojic acid.

The utility of algal sources is based on scale and the complexity of the target product. Macroalgae excel as highly voluminous sources of complex phenolic extracts, particularly phlorotannins, characterized by sophisticated slow-binding kinetics that promise persistent efficacy. The structural complexity (size and hydroxylation count) of molecules like 2-O-(2,4,6-trihydroxyphenyl)-6,6′-bieckol dictates a desirable two-step enzyme isomerization mechanism, providing a roadmap for engineering prolonged pharmacological effects.

In contrast, microalgae are ideally suited for the controlled, industrial production of novel, highly potent single chemical entities, exemplified by potent scytonemin monomer (ScyM) and protein-based pigments, such as C-phycocyanin. The intrinsic sustainability and minimized batch variability afforded by controlled microalgal cultivation make them highly attractive for GMP manufacturing, while their protein components necessitate the use of advanced Nanostructured Lipid Carriers (NLCs) to ensure product stability and bioavailability.

The transition of these compounds from laboratory discovery to commercial products requires concerted effort in three critical areas:

1. Standardization and comparative metrics—Researchers must adopt rigorous, standardized kinetic assays and consistently report Relative Inhibitory Activity (RA) normalized against a positive control to allow for scientifically robust comparison across the highly variable literature [31].
2. Technological integration—The adoption of green extraction technologies, such as Natural Deep Eutectic Solvents (NADESs), is vital for achieving high yield, low cost, and maximal stability of sensitive compounds like phlorotannins during scale-up [51]. Concurrently, microencapsulation and targeted delivery systems are necessary to ensure the clinical efficacy of labile bioactives.
3. Translational safety assessment—Future research must prioritize comprehensive in vivo and ADMET/toxicology evaluations, similar to those performed for stigmasterol, to validate the safety and selectivity of new inhibitors before clinical integration.

Most investigations on potential tyrosinase inhibitors are performed on mushroom (*Agaricus bisporus*) tyrosinase. It is very active, commercially available, cheap, and well-studied. As all tyrosinases possess a conserved active site, mushroom tyrosinase has become a model for human enzymes. Some studies have pointed out that potent mushroom enzyme inhibitors are much weaker than in humans [36]; therefore, in the context of topical or oral application, further tests using human enzymes must be applied. Computational studies for scaffold design using a direct human tyrosinase crystal structure are not feasible, but modern tools like AlphaFold are available to predict protein structures, as discussed in the section considering the tyrosinase active site.

By addressing these translational challenges, marine algal bioactives can fulfil their potential as the next generation of depigmenting and anti-melanogenic agents for the cosmeceutical and pharmaceutical industries.

## Figures and Tables

**Table 1:** Comparative efficacy of selected macroalgal and microalgal tyrosinase inhibitors.

Source Organism (Species)	Compound/ Extract	Chemical Class	IC50 Value (μg/mL or μM)	Kojic Acid Reference IC50 (μM)	Inhibition Type	Ref.
*Cyanobacteria*	scytonemin monomer (ScyM)	apocarotenoid	4.90 µM	11.31 µM	single molecule	[10]
*Ecklonia cava* (brown alga)	total phenolic extract	phlorotannins	4.38 ± 0.08 µg/mL	N/A	non-competitive (extract)	[13]
*Ecklonia cava* (brown alga)	2-phloroeckol	phlorotannin	7.0 ± 0.2 µM	N/A	competitive, slow-binding	[12]
*Eisenia bicyclis* (brown alga)	extract	phlorotannins	4.46 ± 0.52 µg/mL	N/A	non-competitive (extract)	[13]
*Spirulina platensis* (microalga)	phycocyanin	phycobiliprotein	30.88 µg/mL (cell-based)	34.06 µg/mL (Cell-based)	N/A	[20]
*Gracilaria fisheri* (red alga)	stigmasterol (isolated)	sterol	3.38 ± 0.28 µg/mL (cell viability)	N/A	highly selective	[13]

**Table 2:** Mechanistic classification of key algal tyrosinase inhibitors.

Compound/Extract	Source (Algal Division)	Inhibition Type	Key Binding Features /Mechanism	Ref.
2-O-(2,4,6-trihydroxyphenyl)-6,6′-bieckol	Phaeophyceae (*E. cava*)	Competitive, slow-binding	Two-step enzyme isomerisation, extensive H-bonding (Lys79, His85)	[12]
Dieckol	Phaeophyceae (*E. stolonifera*)	Non-competitive	Allosteric binding site, high binding affinity (K_i_ = 15 μM)	[11,32]
Dimeric Bromophenol (Comp. 3)	Rhodophyceae (*S. latiuscula*)	Competitive	Direct active site binding, H-bonding to Arg268 and Per404	[22]
Scytonemin Monomer (ScyM)	Cyanobacteria	Slowly reversible mixed-type	Binds E and E-S complex; phenol moiety indispensable	[10]
Peptide DER	Microalgae (*Spirulina*)	Competitive/active site	H-bonding with His244, His259, His260, Asn260 (MD confirmed)	[39]

**Table 3:** Microalgal classes and associated tyrosinase inhibitory bioactives.

Algal Group (Class)	Representative Species	Primary Active Compound Class	Specific Bioactive Examples	Commercial Advantage/Sustainability Note	Ref.
Cyanobacteria	*Arthrospira platensis* (Spirulina)	Phycobiliproteins	C-Phycocyanin	High biomass productivity, GRAS status, NLC potential for stability	[23]
Cyanobacteria	Marine Cyanobacteria	Apocarotenoids	Scytonemin, Monomer (ScyM)	Ultra-high potency, novel small molecular scaffold	[10]
Chlorophyceae	*Dunaliella tertiolecta*	Carotenoids/phenolics	β-carotene, Zeaxanthin, phenolic acids	Efficient cultivation, dual antioxidant role	[11]
Bacillariophyceae	*Nitzschia* sp.	Phenolics/extracts	Undefined extract	Patented application for melanin inhibition	[27]

## Data Availability

All data included in the manuscript.
